# A K-Band Four-Channel Beamformer with Temperature Compensation Based on 65 nm CMOS Process

**DOI:** 10.3390/mi17040462

**Published:** 2026-04-10

**Authors:** Cetian Wang, Yanning Liu, Xuejie Liao, Fan Zhang, Chun Deng, Ying Liu, Wenxu Sun, He Guan, Deyun Zhou

**Affiliations:** 1 School of Microelectronics, Northwestern Polytechnical University, Xi’an 710129, China; wangct@ganide.com (C.W.); sunwenxu@mail.nwpu.edu.cn (W.S.); dyzhou@nwpu.edu.cn (D.Z.); 2 Chengdu Ganide Technology Company, Ltd., Chengdu 610220, China; liuyn@ganide.com (Y.L.); liaoxuejie@foxmail.com (X.L.); zhangfan_uestc@163.com (F.Z.); dengc@ganide.com (C.D.); liuy@ganide.com (Y.L.)

**Keywords:** K-band, active phased array, beamformer, root mean square, 65 nm CMOS

## Abstract

This paper presents a K-band four-channel phased array beamformer with temperature compensation in 65 nm CMOS for 5G and satellite communications. The beamformer includes a four-way power divider/combiner, four RF channels, and digital control circuits. Each RF channel comprises a receive chain, a transmit chain, and a pair of receive/transmit (TX/RX) single-pole double-throw (SPDT) switches. The receive chain consists of a low-noise amplifier (LNA), a six-bit reflective-type phase shifter (RTPS), a drive amplifier (DA), two temperature-compensation attenuators (TCAs), and a six-bit attenuator (ATT); the transmit chain integrates a power amplifier (PA), two TCAs, a six-bit RTPS, a DA, and a six-bit ATT. Measurements show the chip exhibits 0–4.5 dB gain, noise figure (NF) < 7.8 dB, root mean square (RMS) phase error < 3.5°, and RMS gain error < 0.4 dB in receive mode operating in 19–23 GHz. In transmit mode operating in 21–23 GHz, it provides 6–10 dB gain range, RMS phase error < 3.4°, RMS gain error < 0.25 dB, and output power at 1 dB compression point (OP1dB) > 6.5 dBm. In addition, the receive and transmit gain variations are within 0.8 dB and 0.4 dB, respectively, when temperature ranges from −55 °C to 85 °C. With a compact footprint of 3.5 × 4.8 mm^2^, the beamformer consumes 110 mW (receive) and 190 mW (transmit) DC power per channel.

## 1. Introduction

Active phased-array beamforming is a key enabling technology for 5G and satellite communications [1,2,3,4,5,6]. These systems comprise multiple beamformer elements that must satisfy strict miniaturization, cost-effectiveness, and high-performance requirements. Precise amplitude and phase tuning allows these beamformers to generate directional, electronically steerable beams [7,8,9], which mitigate inherent free-space path loss to extend communication range and enable spatial multiplexing for improved communication efficiency [10,11].

In the past decade, a number of beamformers based on III–V compound and silicon processes have been reported in [12,13,14,15]. Ref. [12] presents a 32–38 GHz GaAs four-channel beamformer based on a 3D stacked-chip process, with RMS phase error < 5.5° and RMS gain error < 0.3 dB. The 3D stacked chip is formed by vertically integrating a CMOS control chip, a GaAs carrier, and a GaAs RF chip through heterogeneous integration. Compared with GaAs processes, silicon-based beamformers are more extensively adopted in phased arrays due to their cost efficiency and high integrability. For 5G beamforming systems, Ref. [13] implements a Ka-band four-channel bidirectional beamformer in 65 nm CMOS, which achieves a measured RMS gain error < 0.5 dB at 26–32 GHz but suffers from relatively low phase-shift resolution with RMS phase error approaching 7°. To achieve high-resolution phase/amplitude control, Ref. [14] designed a 32.2–38.2 GHz broadband four-channel beamformer with an embedded three-winding transformer using 65 nm CMOS, exhibiting measured RMS phase error < 1.66° and RMS gain error < 0.18 dB. Notably, it is essential to guarantee the reliability of phased-array systems across a broad temperature range, as thermal variations commonly result in the degradation of signal integrity. To alleviate this effect, various temperature-compensation (TC) strategies have been developed, such as proportional-to-absolute-temperature (PTAT) voltage-based amplifiers [16], TC-aided variable gain amplifiers (VGAs) [17], and temperature-regulated voltage-variable attenuators (VVAs) [18], which are implemented to sustain a constant gain performance of the circuit. Recently, Ref. [15] reported a temperature-compensated Ku-band SiGe BiCMOS four-beam phased-array receiver that reduces gain variations against temperature fluctuations with low attenuation and small relative phase variations; however, this design only includes receive channels. As active phased-array technology evolves, the demand for beamformers with multi-channel, multifunctional integration (including temperature compensation) and a compact footprint is increasing.

In this paper, a K-band four-channel phased-array beamformer implemented in 65 nm CMOS technology is presented. The proposed beamformer, featuring a compact area of only 3.5 × 4.8 mm^2^, integrates multiple functionalities including transmit–receive amplification, amplitude attenuation, phase shifting, temperature compensation, and digital circuit control. The remainder of this paper is organized as follows: First, the architecture of the four-channel beamformer is described. This is followed by design and analysis of the key building blocks. Finally, the measured performances of the fabricated beamformer are presented and discussed, prior to the conclusion.

## 2. Architecture Design Overview

**Figure 1:**
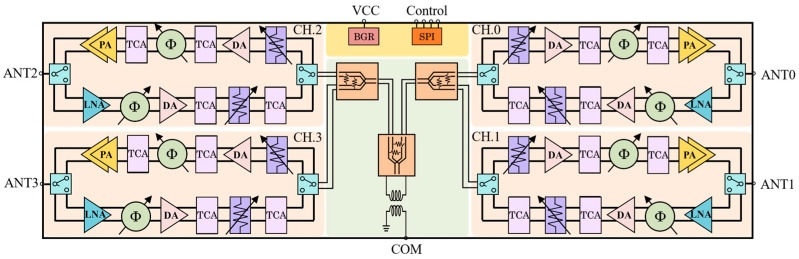
Block diagram of the presented four-channel beamformer.

Figure 1 presents the block diagram of the developed phased-array beamformer. The chip incorporates a four-way power divider/combiner, four RF channels (designated as CH.0, CH.1, CH.2, and CH.3), an analog circuit module and digital control circuitry. Each RF channel encompasses a receive chain, a transmit chain, and a pair of TX/RX SPDT switches. In detail, the receive chain is configured with an LNA, a six-bit RTPS, a DA, two TCAs, and a six-bit ATT; the transmit chain, on the other hand, is structured with a PA, two TCAs, a six-bit RTPS, a DA, and a six-bit ATT.

The four-way power divider/combiner, consisting of two two-way Wilkinson power dividers, serves to divide a single RF input (marked COM) into four channel outputs during transmit mode, or to combine four channel inputs into a single output during receive mode. The six-bit RTPS achieves a total controllable phase shift of 354.375° with a phase step of 5.625°, enabling precise phase state tuning. Correspondingly, the six-bit ATT realizes a total controllable attenuation of 23.5 dB with an attenuation step of 0.5 dB, facilitating accurate amplitude level adjustment. The LNA placed at the front end of the receive chain is characterized by high gain and low NF, which is critical for enhancing the signal-to-noise ratio (SNR) of the received signal. The PA located at the output stage of the transmit chain is capable of delivering adequate output power to meet the system requirements. Moreover, the DAs integrated in both the receive and transmit chains function to compensate for the insertion losses incurred by the six-bit RTPS and six-bit ATT, and to provide the necessary driving power for the PA in the transmit chain. Positioned at the interfaces of the receive and transmit chains in each channel, the TX/RX SPDT switches facilitate reliable RF path switching between transmit and receive modes. The TCAs embedded in the transmit and receive chains are specifically designed to mitigate the gain variations of the amplifiers caused by temperature fluctuations. A fully differential architecture is adopted for the channel design to effectively suppress common-mode noise and minimize inter-channel crosstalk [19]. Furthermore, to facilitate chip control and provide stable DC bias voltages, a digital serial peripheral interface (SPI) circuit integrated with a low dropout regulator (LDO) and a bias circuit are implemented on the chip.

## 3. Circuit Design

**Figure 2:**
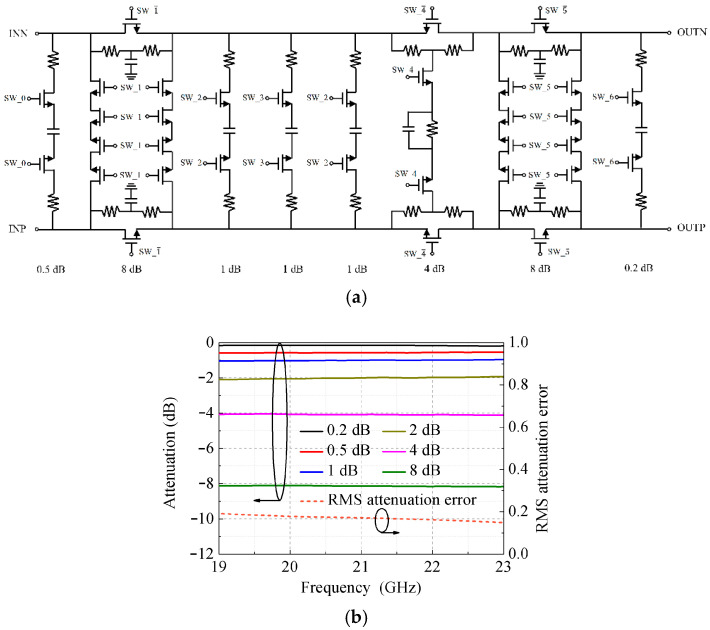
Schematic and simulated results of the digital attenuator. (**a**) Schematic circuit. (**b**) Simulated attenuations and RMS attenuation error.

Figure 2a depicts the circuit topology of a 6-bit 23.5 dB ATT. This ATT comprises a cascade of fully differential switched π/T-type attenuation cells [20,21,22,23], offering attenuation values of 0.2 dB, 0.5 dB, 1 dB, 2 dB, 4 dB, and two 8 dB increments. The 0.2 dB cell serves as a tuning unit to compensate for attenuation deviations. A simplified T-type configuration is adopted for the 0.2 dB, 0.5 dB, 1 dB, and 2 dB cells to achieve a compact layout; notably, the 2 dB cell is implemented with a two-stage series-connected 1 dB attenuation structure. For the 4 dB cell, a bridge-T topology integrated with a parallel tail capacitor is utilized, taking advantage of its intrinsic characteristics of low insertion loss and favorable impedance matching. In this design, the parallel tail capacitor is deliberately introduced to mitigate the parasitic phase shift induced by the attenuator. For the two 8 dB cells, a π-type topology is selected based on its capability of providing a large attenuation range and low parasitic phase shift. All attenuation cells are independently optimized and are arranged in an appropriate sequence. Importantly, the two 8 dB cells are spaced apart to enhance the impedance matching of the ATT.

Figure 2b depicts the simulated attenuation responses of the 6-bit attenuator (ATT), demonstrating that these six typical attenuation states agree well with the ideal attenuation values. Furthermore, each attenuation state exhibits nearly identical attenuation magnitude across the operating frequency band, thereby achieving flat attenuation characteristics. The RMS attenuation error is computed as follows:

(1) $$A_{\Delta ,RMS}=\sqrt{\frac{1}{N-1}\sum_{k=2}^{k=N}{\left|\Delta A_{k}\right|}^{2}}$$

where $\Delta A_{k}$ (*k* = 2, …, *N* − 1, *N* = 48) denotes the relative attenuation error for the *k*-th attenuation state. The RMS attenuation error calculated over all 48 attenuation states of the ATT is less than 0.18 dB (at 19 GHz) across the full operating frequency band, and decreases with increasing frequency.

**Figure 3:**
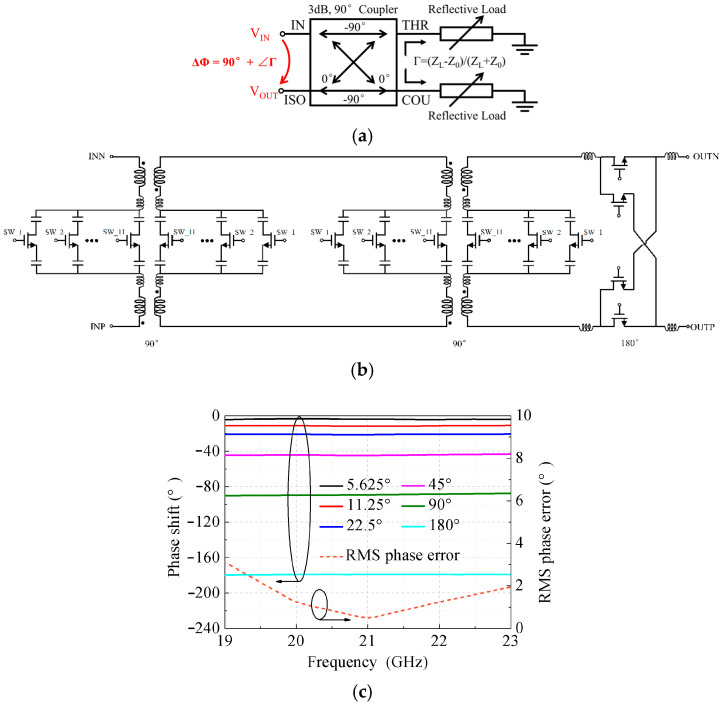
Schematic and simulated results of the RTPS. (**a**) Topology of the single-ended RTPS circuit. (**b**) Schematic circuit. (**c**) Simulated phase shifts and RMS phase error.

The reflective-type phase shifter (RTPS) can realize large phase shifts, high linearity, and fine phase-shift resolution [23,24,25]. Figure 3a shows the circuit topology of a conventional RTPS architecture [24]. When the input signal is fed into the input port, two signals with 90° phase difference are produced by the 90° coupler. Subsequently, these two signals are reflected by the identical reflective loads at the through and couple ports, and generate an output signal with the desired phase shift at the isolation port. The phase shift θ can be expressed as follows:

(2) $$\theta =-90-2\tan^{-1}\left(\frac{X_{L}}{Z_{0}}\right)$$

where *X_L_* is the reflective load impedance and *Z*_0_ is the characteristic impedance of the 90° coupler. It is evident that the output phase shift can be achieved by tuning the reflective load impedance. As *X_L_* varies from $X_{L}^{min}$ to $X_{L}^{max}$, the phase-shift range of the RTPS can be derived as follows:

(3) $$\Delta \theta =\theta_{max}-\theta_{min}=2\tan^{-1}\left(\frac{X_{L}^{max}}{Z_{0}}\right)-2\tan^{-1}\left(\frac{X_{L}^{min}}{Z_{0}}\right)$$

Based on the phase-shifting theory demonstrated above, a 6-bit reflective-type phase shifter (RTPS) employing a reflective-load configuration is designed, as illustrated in Figure 3b, to realize phase shifts of 5.625°, 11.25°, 22.5°, 45°, 90°, and 180°. This topology consists of a 90° hybrid coupler, a pair of reflective loads, and inductive elements. Among them, the 90° hybrid coupler is realized using a transformer that exhibits wideband performance and a small footprint, and it is employed to divide the input signal into two signals with a phase difference of 90°. The two reflective loads are constructed using switched capacitor arrays, where the required phase shift range is obtained by controlling the on/off states of the switches. In addition, the introduction of a series inductor before the capacitor arrays effectively enhances the phase-tuning resolution of the RTPS.

Figure 3c presents the simulated typical phase states of the RTPS, demonstrating that flat phase-shift responses are achieved. Furthermore, the phase-shifting accuracy across all 64 states can be quantified by the RMS phase error, which is defined in Equation (4):

(4) $$\theta_{\Delta ,RMS}=\sqrt{\frac{1}{N-1}\sum_{k=2}^{k=N}{\left|\Delta \theta_{k}\right|}^{2}}$$

where $\Delta \theta_{k}$ (*k* = 2, …, N − 1, N = 64) represents the relative phase error of the *k*-th phase shifting state. As shown in Figure 3c, the computed RMS phase error is below 3.1° across the entire operating bandwidth and reaches a minimum of approximately 0.5° near 21 GHz. As the frequency deviates from the center frequency, the relative phase error increases, leading to a slight degradation in phase-shifting accuracy at both the lower and higher frequency ends.

**Figure 4:**
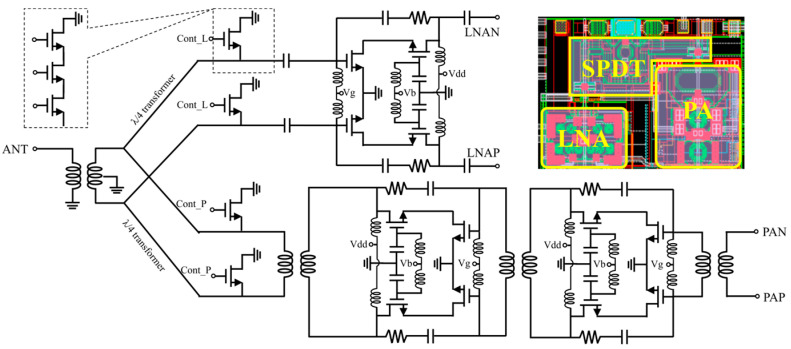
Schematic of the proposed LNA, PA and TX/RX switch.

Figure 4 illustrates the circuit schematic of a co-integrated LNA, PA, and TX/RX SPDT switch. The differential output ends of the PA and differential input ends of the LNA are directly interfaced with an antenna balun. The TX/RX SPDT switch serves to alternate between the receiving and transmitting paths. Since the isolation performance of the beamformer can be achieved by enabling or disabling the bias voltages of the LNA and PA, the SPDT switch is permitted to trade off a small amount of isolation to minimize its insertion loss, which is achieved by adopting a conventional parallel topology [26,27]. For the purpose of further enhancing the voltage withstand capability and linearity of the switch, a 3-series NMOS transistor topology is implemented. Correspondingly, an identical TX/RX SPDT switch is integrated at the other port of the channel that is connected to the four-way power divider. The PA and LNA are both designed with a differential cascode amplification architecture: specifically, the LNA is constructed using a 1-stage cascode amplification structure, whereas the PA employs a 2-stage cascode amplification structure owing to its advantages of high gain and large output voltage swing, which contributes to the improvement of output power performance. To optimize wideband characteristics and gain flatness, an inductive source degeneration technique and an RC feedback network are incorporated [28,29,30,31]. Notably, the DAs demonstrated in Figure 1 employ the same amplification structure as the LNA. The partial layout views of the SPDT, LNA, and PA circuits are displayed in the top inset of Figure 4.

**Figure 5:**
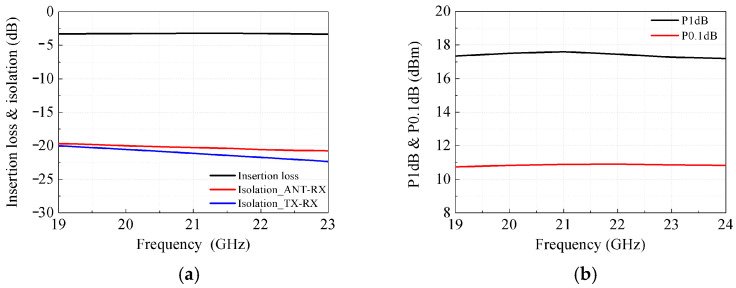

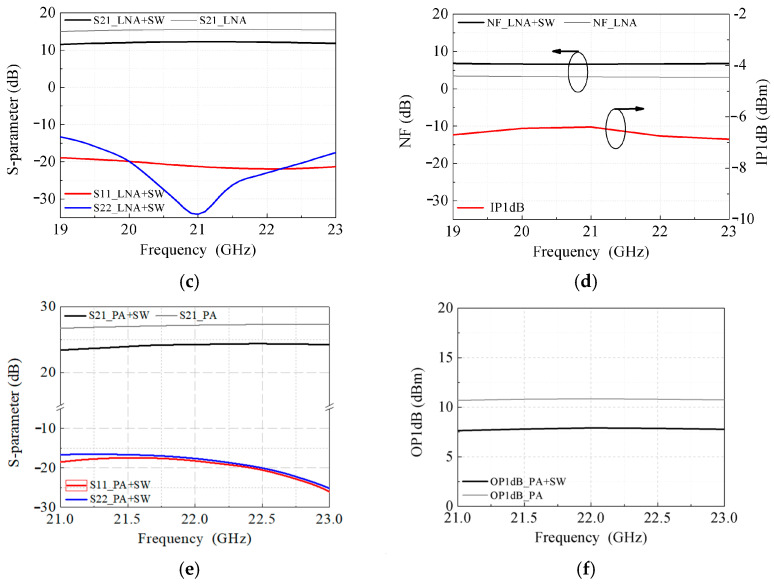
Simulated results of the SPDT switch, LNA and PA. (**a**) Simulated insertion loss, isolation and (**b**) OP1dB, OP0.1dB of the SPDT switch. (**c**) Simulated S-parameters and (**d**) NF, IP1dB of the LNA cascaded with the SPDT switch. (**e**) Simulated S-parameters and (**f**) OP1dB of the PA cascaded with the SPDT switch.

In Figure 5a, the insertion loss of the SPDT switch ranges from −3.2 dB to −3.4 dB across the operating frequency band. The isolation between ANT and RX, as well as between TX and RX ports, is better than −20 dB. The simulated P1dB of the switch in Figure 5b is greater than 17 dBm, and the P0.1dB exceeds 10 dBm, revealing good linearity of this SPDT switch. The simulated performances of the proposed LNA and PA with and without the SPDT switch are illustrated in Figure 5c–f. The cascaded LNA and SPDT switch exhibits a gain of 11.5–12.3 dB, a noise figure (NF) of 6.5–6.7 dB, an input of 1 dB compression point (IP1dB) above −6.87 dBm, and input/output return losses better than 13.5 dB, as depicted in Figure 5c,d. As the SPDT switch is positioned before the LNA, its insertion loss degrades the NF and gain by 3.2–3.6 dB relative to the standalone LNA. As shown in Figure 5e,f, the combined PA and SPDT switch delivers a gain of 23.4–24.4 dB, and an output of 1 dB compression point (OP1dB) exceeding 7.6 dBm, with input and output return losses superior to 16.5 dB. The SPDT switch cascaded at the PA output leads to a 3–3.3 dB degradation in both output power and gain compared with the standalone PA.

**Figure 6:**
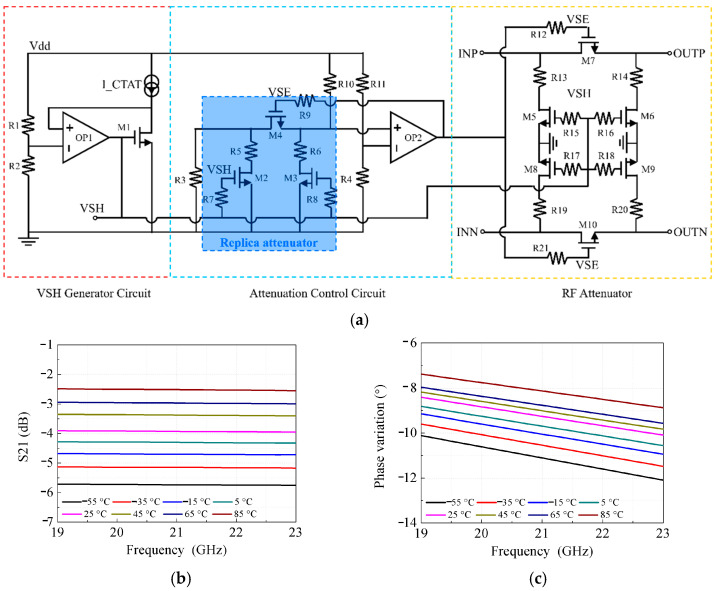
Schematic circuit and simulated insertion loss and phase with different temperatures. (**a**) Schematic circuit. (**b**) Simulated insertion loss and (**c**) phase variation against frequency with temperature varying from −55 °C to 85 °C.

The TCA is developed to compensate for the temperature-induced gain drift in amplifiers. The schematic topology of the proposed TCA is illustrated in Figure 6a, which consists of a control-voltage-of-shunt-MOSFET (VSH) generator circuit, an attenuation control circuit, and an RF attenuator. The voltages VSH and VSE are utilized to bias the series and shunt MOSFETs in both the replica attenuator and the RF attenuator, where all MOSFETs operate in the triode region. A complementary-to-absolute-temperature (CTAT) voltage VSH is generated by the VSH generator based on the ICTAT principle, and can be directly applied to bias the gates of the shunt MOSFETs. The attenuation control circuit, comprising a replica attenuator and a feedback loop, is designed to automatically compensate for the equivalent impedance variation of the attenuator, and the control-voltage-of-series MOSFET (VSE) is generated accordingly. As a result, the desired attenuation level of the RF attenuator is achieved by applying the same VSE and VSH biases, while good input/output impedance matching is simultaneously maintained.

Figure 6b,c show the simulated S-parameters over the operating frequency band at temperatures ranging from −55 °C to 85 °C in 20°C steps. The TCA exhibits an insertion loss of 4 dB at 25 °C, with temperature compensations of 1.5 dB and 1.7 dB at 85 °C and −55 °C, respectively. The integration of two TCAs in both the TX and RX channels provides a strong gain compensation capability exceeding 6 dB over the entire temperature range. As illustrated in Figure 6c, the phase responses of S21 vary with temperature, shifting from −12° to −7.5° across the full temperature range.

## 4. Measurement Results

**Figure 7:**
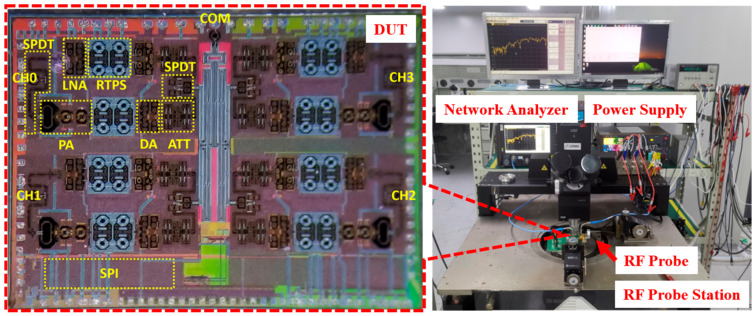
Measurement setup and microphotograph of the beamformer.

The beamformer is fabricated in a 65 nm CMOS process. All measurements were conducted using an RF probe station, network analyzer, and power supply, as shown in Figure 7. The chip microphotograph is presented, with a total area of 4.5 × 3.5 mm^2^ and a thickness of 200 μm. The chip is biased at 3.3 V and 2.0 V for optimal performance. The measured DC power consumption per channel is 189 mW in the transmit mode and 110 mW in the receive mode.

**Figure 8:**
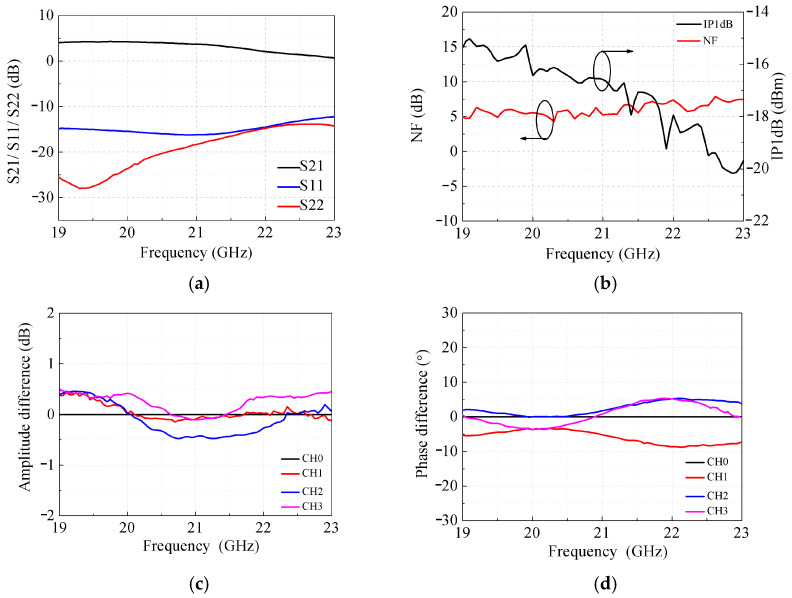

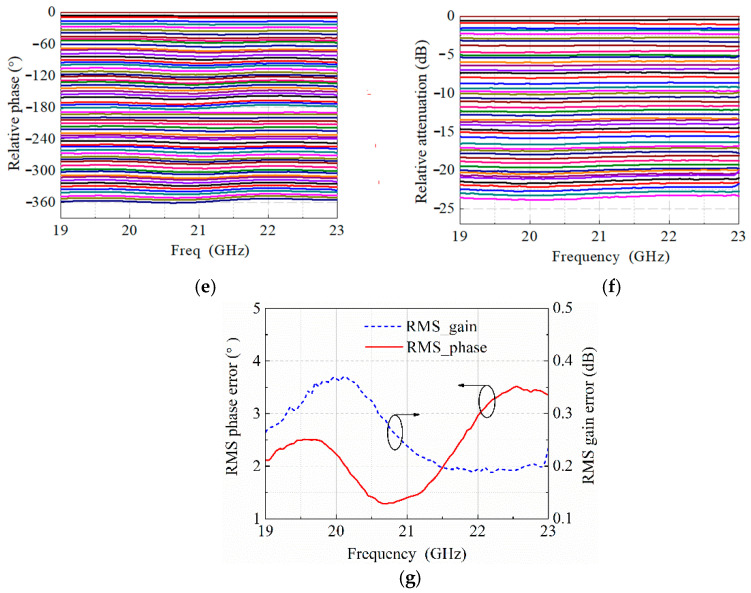
Measured results of the beamformer in receive mode. (**a**) S-parameters. (**b**) NF and IP1dB. (**c**) Amplitude differences and (**d**) phase differences of gain. (**e**) Phase shifts in 64 states. (**f**) Attenuations in 48 states. (**g**) RMS phase and gain errors.

Figure 8 demonstrates the measured performance of the beamformer in receive mode. Notably, as shown in Figure 8a,b, the beamformer achieves a receive gain of 0–4.5 dB, an NF below 7.8 dB, and an IP1dB exceeding −20 dBm over the 19–23 GHz frequency range, along with input and output return losses better than 12 dB. The inter-channel consistency of the receive gain, depicted in Figure 8d with CH.0 as the reference, is superior, with maximum amplitude and phase differences of less than 1 dB and 15°, respectively. The measured relative phase shifts and attenuations across all receive states (Figure 8e,f) exhibit remarkable flatness across the entire operating band. To validate the precision of phase and gain control, Figure 8g presents the RMS phase and gain errors, which are constrained to below 0.4 dB and 3.5°, respectively.

**Figure 9:**
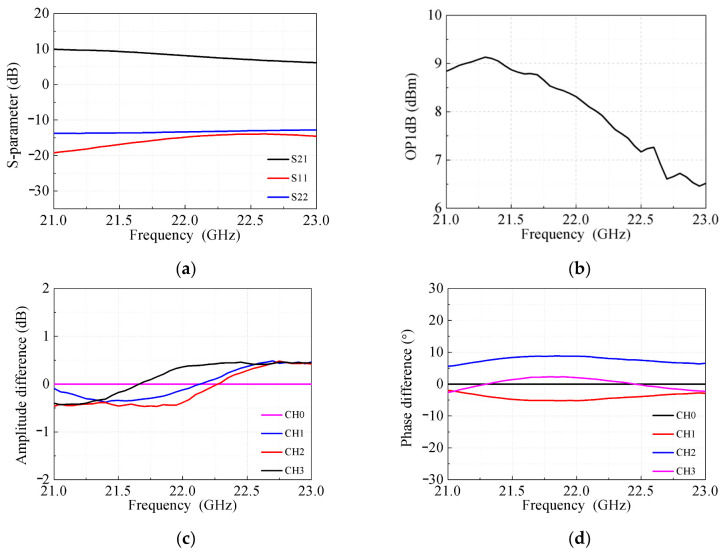

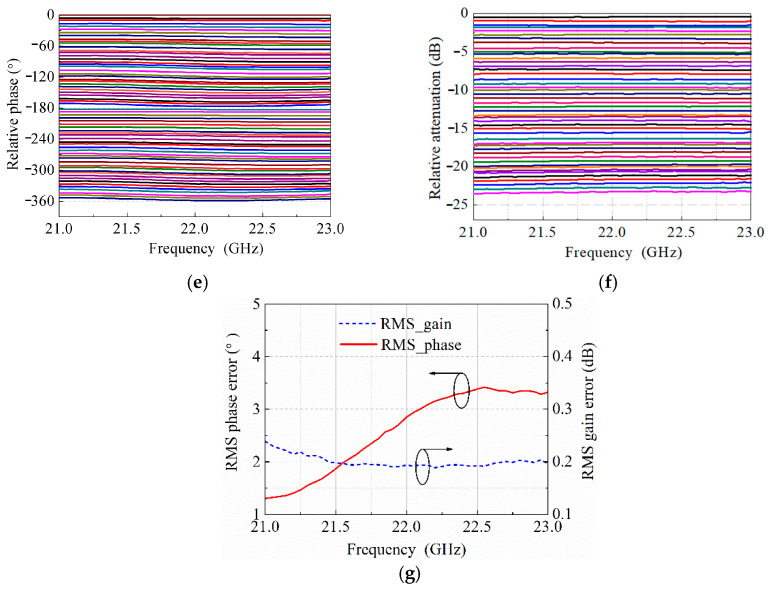
Measured results of the beamformer in transmit mode. (**a**) S-parameters. (**b**) OP1dB. (**c**) Amplitude differences and (**d**) phase differences of gain. (**e**) Phase shifts in 64 states. (**f**) Attenuations in 48 states. (**g**) RMS phase and gain errors.

In transmit mode, the measured performance demonstrated in Figure 9 is equally impressive. Within the 21–23 GHz band, Figure 9a,b show a transmit gain of 6–10 dB, an OP1dB higher than 6.5 dBm, and input/output return losses better than 12 dB. The inter-channel uniformity of the transmit gain shown in Figure 9c,d is excellent, with maximum amplitude and phase differences of less than 1 dB and 15°. The relative phase shifts and attenuations across all transmit states depicted in Figure 9e,f maintain flat responses, and the RMS gain and phase errors shown in Figure 9g are as low as 0.25 dB and 3.4°, respectively, confirming high-precision transmit gain and phase shift control.

**Figure 10:**
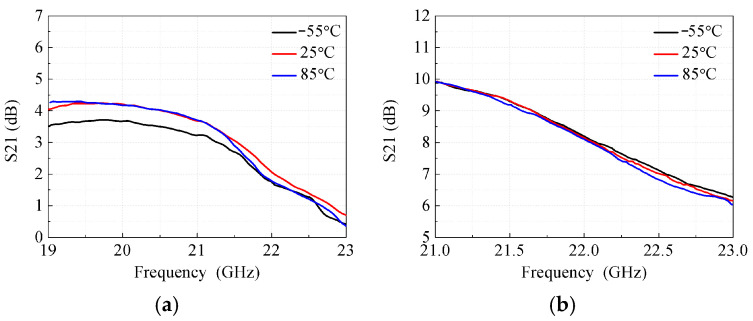
Measured gain of the beamformer against different temperatures. (**a**) Measured receive gain. (**b**) Measured transmit gain.

Temperature stability is a critical performance metric, as shown in Figure 10a,b, which present the receive and transmit gains under varying temperatures. When the temperature ranges from −55 °C to 85 °C, the receive gain variation is limited to less than 0.8 dB, and the transmit gain variation is within 0.4 dB. This outstanding temperature stability ensures reliable operation of the beamformer under extreme environmental conditions.

Finally, Table 1 presents a comprehensive performance comparison between the proposed work and state-of-the-art beamformers. Notably, in contrast to previous works, the developed four-channel beamformer exhibits competitive performance metrics, including RMS gain error, RMS phase shift error, temperature compensation range, and power consumption.

## 5. Conclusions

In this work, a K-band four-channel phased-array beamformer is designed, implemented, and thoroughly characterized using a 65 nm CMOS process. Each channel of the proposed beamformer incorporates an LNA, a PA, a pair of TX/RX SPDT switches, two DAs, two six-bit RTPSs, two six-bit ATTs, and four TCAs. Comprehensive measured results verify that in receive mode, the chip achieves a 0–4.5 dB gain, an NF below 7.8 dB, an RMS phase error of less than 3.5°, and an RMS gain error of less than 0.4 dB. In transmit mode, it delivers a 6–10 dB gain range, an RMS phase error within 3.4°, an RMS gain error of 0.25 dB, and an OP1dB of 6.5 dBm. Over −55 °C to 85 °C, the receive gain variation is less than 0.8 dB, and the transmit gain variation is within 0.4 dB. Owing to its superior performance, the developed beamformer holds significant potential for practical applications in 5G and satellite communication systems.

## Figures and Tables

**Table 1:** Comparison to previously reported beamformers.

Ref.	[12]		[13]		[14]		[15]	This Work	
Process	90 nm GaAs+ 180 nm CMOS		65 nm CMOS		65 nm CMOS		130 nm SiGe BiCMOS	65 nm CMOS	
Integration	4CH TRX		4CH TRX		4CH TRX		8CH RX	4CH TRX	
	RX	TX	RX	TX	RX	TX		RX	TX
Frequency (GHz)	32–38		26–32		32.2–38.2		10.7–12.7	19–23	21–23
Minimum Gain (dB)	22	25	4	10	31.5	31.5	21.2-	0	6
NF (dB)	3.5	-	9.3	-	5.5	-	2.5	7.8	-
Gain Tuning Range/Step (dB)	-		31/1		31.5/0.5		15.5/0.5	23.5/0.5	
RMS Gain Error (dB)	-		0.5		0.18		0.71	0.4	0.25
Phase Step (°)	5.625 (6-bit)		11.25 (5-bit)		5.625 (6-bit)		5.625 (6-bit)	5.625 (6-bits)	
RMS Phase Error (°)	5.5		7		1.66		2.7	3.5	3.4
RX IP1dB (dBm)	-		−22		-		−38.9	−20	
TX OP1dB (dBm)	27		−2		15.8		-	6.5	
Pdc/Channel (mW)	-	-	228	380	130	550	115	109.7	189
Temperature compensation range (°C)	-		-		-		−40–85	−55–85	
Chip Area (mm^2^)	33.64		4.92		9.24		31.86	16.8	

## Data Availability

The presented data in this paper are available on request from the corresponding author.
